# The Rabies Virus L Protein Catalyzes mRNA Capping with GDP Polyribonucleotidyltransferase Activity

**DOI:** 10.3390/v8050144

**Published:** 2016-05-21

**Authors:** Minako Ogino, Naoto Ito, Makoto Sugiyama, Tomoaki Ogino

**Affiliations:** 1 Department of Molecular Biology and Microbiology, Case Western Reserve University School of Medicine, Cleveland, OH 44106, USA; minako.ogino@case.edu; 2 Laboratory of Zoonotic Diseases, Faculty of Applied Biological Sciences, Gifu University, 1-1 Yanagido, Gifu 501-1193, Japan; naotoito@gifu-u.ac.jp (N.I.); sugiyama@gifu-u.ac.jp (M.S.); 3 The United Graduate School of Veterinary Sciences, Gifu University, 1-1 Yanagido, Gifu 501-1193, Japan

**Keywords:** rabies virus, L protein, mRNA capping, GDP polyribonucleotidyltransferase

## Abstract

The large (L) protein of rabies virus (RABV) plays multiple enzymatic roles in viral RNA synthesis and processing. However, none of its putative enzymatic activities have been directly demonstrated *in vitro*. In this study, we expressed and purified a recombinant form of the RABV L protein and verified its guanosine 5′-triphosphatase and GDP polyribonucleotidyltransferase (PRNTase) activities, which are essential for viral mRNA cap formation by the unconventional mechanism. The RABV L protein capped 5′-triphosphorylated but not 5′-diphosphorylated RABV mRNA-start sequences, 5′-AACA(C/U), with GDP to generate the 5′-terminal cap structure G(5′)ppp(5′)A. The 5′-AAC sequence in the substrate RNAs was found to be strictly essential for RNA capping with the RABV L protein. Furthermore, site-directed mutagenesis showed that some conserved amino acid residues (G1112, T1170, W1201, H1241, R1242, F1285, and Q1286) in the PRNTase motifs A to E of the RABV L protein are required for cap formation. These findings suggest that the putative PRNTase domain in the RABV L protein catalyzes the rhabdovirus-specific capping reaction involving covalent catalysis of the pRNA transfer to GDP, thus offering this domain as a target for developing anti-viral agents.

## 1. Introduction

Rabies virus (RABV), a member of the *Lyssavirus* genus belonging to the *Rhabdoviridae* family in the order *Mononegavirales*, is a causative agent of classic rabies, a fatal neurological disease, in humans and animals [1,2]. Rabies kills more than 50,000 people each year worldwide, especially in developing countries [3]. Furthermore, RABV-related lyssaviruses (e.g., Mokola virus, Duvenhage virus, European bat lyssaviruses 1 and 2, Australian bat lyssavirus) are known to cause rabies-like diseases in humans [4]. Therefore, it is important to develop therapeutic targets against these lyssaviruses.

RABV possesses an 11.9 kb negative-strand RNA genome, which contains five genes encoding nucleocapsid (N), phospho- (P), matrix (M), glyco- (G), and large (L) proteins [1,2]. RABV particles deliver ribonucleoprotein complexes into the cytoplasm of host cells, where RNA-dependent RNA polymerase (RdRp) complexes composed of the L and P proteins synthesize viral mRNAs from the genomic RNA template encapsidated with the N proteins. However, the inability to establish efficient *in vitro* transcription systems [5,6] has hampered progress in understanding the molecular mechanisms of RABV mRNA biosynthesis.

Structural homology of the L protein (2127 amino acids) of RABV to that of vesicular stomatitis virus (VSV), a well-characterized rhabdovirus belonging to the *Vesiculovirus* genus, suggests that it catalyzes all enzymatic reactions required for viral RNA synthesis and processing (mRNA 5′-capping, cap methylation, and 3′-polyadenylation) [7,8,9]. According to the model proposed from intensive studies using VSV [10,11,12,13], the RABV RdRp complex starts transcription at the 3′-end of the RNA genome to synthesize the short uncapped leader RNA, and sequentially produces five mRNAs from the tandemly aligned genes (3′-*N*-*P*-*M*-*G*-*L*-5′) by the stop-start mechanism. The five RABV mRNAs appear to be 5′-capped, methylated, and 3′-polyadenylated during transcription, as in the case of VSV mRNAs [14,15].

We found that the L proteins of vesiculoviruses, VSV (2109 amino acids) and Chandipura virus (CHPV, 2092 amino acids) catalyze a unique mRNA capping reaction [16,17,18,19], which is strikingly different from eukaryotic mRNA capping [20]. The first enzymatic activity involved in vesiculoviral mRNA capping is the guanosine 5′-triphosphatase (GTPase) activity associated with the L protein that hydrolyzes GTP into GDP and inorganic phosphate (P_i_) [16,17]. GDP, in turn, serves as a 5′-monophospho-RNA (pRNA) acceptor for the subsequent reaction with a novel enzyme, GDP polyribonucleotidyltransferase (PRNTase; EC 2.7.7.88) [16,17]. The putative PRNTase domain in the VSV L protein specifically recognizes the 5′-triphosphorylated VSV mRNA-start sequence (5′-AACAG) as a pRNA donor to form a covalent enzyme-pRNA complex (L-pRNA intermediate) with concomitant release of inorganic pyrophosphate (PP_i_) [16,19]. Finally, pRNA is transferred from the intermediate to GDP to generate the 5′-terminal cap structure G(5′)ppp(5′)A on RNA [19]. The PRNTase activity of the VSV L protein is also essential for accurate stop-start transcription to produce full-length mRNAs *in vitro* and virus gene expression and propagation in host cells [21,22].

Non-segmented negative-strand RNA viral L proteins share a general organization of functional domains with six highly conserved regions (called blocks I–VI) [7]. Conserved amino acid residues in block V of the VSV and CHPV L proteins have been identified as important or essential for mRNA capping [18,19,22,23]. Key amino acid residues (G1100, T1157, W1188, H1227, R1228, F1269, and Q1270) required for the L-pRNA intermediate formation in the PRNTase reaction were mapped within five conserved sequence motifs (called motifs A–E) of the putative PRNTase domain (block V) of the VSV L protein [19,22]. A lone pair of electrons formed on the *N*^ε2^ position of the catalytic histidine residue at position 1227 (H1227) in motif D (also called the histidine-arginine (HR) motif) of the VSV L protein was suggested to nucleophilically attack the α-phosphorus in the 5′-triphosphate group of RNA to form the covalent L-pRNA intermediate [19]. All these motifs are conserved in the putative PRNTase domains of rhabdoviruses (except for novirhabdoviruses) and other non-segmented negative-strand RNA viruses belonging to the *Paramyxoviridae* (e.g., measles), *Filoviridae* (e.g., Ebola), *Bornaviridae* (e.g., Borna disease), and *Nyamiviridae* (e.g., Nyamanini) families [9,19,22,24]. In the recently solved three-dimensional structure of the VSV L protein [25], these key residues in motifs A–E were found to be localized in close proximity to form the PRNTase active site [22]. Amino acid residues involved in GDP binding or pRNA transfer are currently unknown.

In this study, to verify the putative enzymatic activities of the RABV L protein, we expressed and purified its recombinant protein. We demonstrated for the first time that the recombinant RABV L protein catalyzes RNA capping with the GTPase and PRNTase activities. The PRNTase domain in the RABV L protein specifically capped the 5′-end of the lyssaviral mRNA-start sequence 5′-AACA(C/U), in which the 5′-AAC sequence is strictly required for the substrate activity.

## 2. Materials and Methods

### 2.1. Expression and Purification of the Recombinant RABV L Protein

The recombinant wild-type (WT) and mutant L proteins of RABV (RC-HL strain, [26]) were expressed as C-terminal polyhistidine (His)-tagged forms (2127 plus 10 [GTHHHHHHHH] amino acids) in Sf21 insect cells (5 × 10^7^ cells) by the Bac-to-Bac baculovirus expression system (Invitrogen, Carlsbad, CA, USA), and purified by chromatography using nickel-nitrilotriacetic acid agarose (Qiagen, Hilden, Germany), as described for the recombinant VSV L protein [27]. Typically, 10–20 μg of the proteins were obtained. To generate mutant *L* genes, site-directed mutagenesis was performed using the Quick Change Lightning mutagenesis kit according to the manufacturer’s instruction (Agilent Technologies, Santa Clara, CA, USA).

### 2.2. Sodium Dodecyl Sulfate Polyacrylamide Gel Electrophoresis (SDS-PAGE) and Western Blotting

Sodium dodecyl sulfate polyacrylamide gel electrophoresis (SDS-PAGE) and Western blotting were performed as described previously [16]. Mouse anti-His-tag monoclonal antibody (GenScript, Piscataway, NJ, USA), horseradish peroxidase-conjugated goat anti-mouse IgG polyclonal antibody (Santa Cruz Biotechnology, Dallas, TX, USA), and ECL detection system (GE Healthcare Life Sciences, Pittsburgh, PA, USA) were used to detect His-tagged proteins.

### 2.3. RNA Capping and GTP Hydrolysis Assays

Five-nucleotide oligo-RNA substrates with different sequences were synthesized with T7 RNA polymerase using partially double-stranded oligo-DNAs as templates as described previously [27]. *In vitro* RNA capping was carried out with the recombinant RABV L protein (0.1 μg, WT or mutant) using 5 μM oligo-RNA and 0.25 μM [α-^32^P]GDP or [α-^32^P]GTP (1.5–2 × 10^3^ cpm/fmol) as substrates according to the method for the VSV L protein [27]. The vaccinia virus capping assay was performed with 50 μM [α-^32^P]GTP (1.5–2 × 10^3^ cpm/pmol), 1 μM oligo-RNA, and three units of vaccinia virus capping enzyme (Cellscript, Madison, WI, USA) as described [16]. Calf intestine alkaline phosphatase and nuclease P_1_-resistant products were analyzed together with standard nucleotides (GMP, GDP, GTP, G(5′)ppp(5′)A (New England Biolabs, Ipswich, MA, USA), and/or G(5′)ppp(5′)G (New England Biolabs)) by thin layer chromatography on a polyethyleneimine-cellulose plate (EMD Millipore, Billerica, MA, USA) (PEI-cellulose TLC) as described [27]. The GTP hydrolysis assay was performed with 0.1 or 0.2 μg of the recombinant RABV L protein using 0.25 μM [γ-^32^P]GTP (2 × 10^4^ cpm/pmol) as described [17,19].

## 3. Results

### 3.1. The RABV L Protein Catalyzes Unconventional RNA Capping

**Figure 1:**
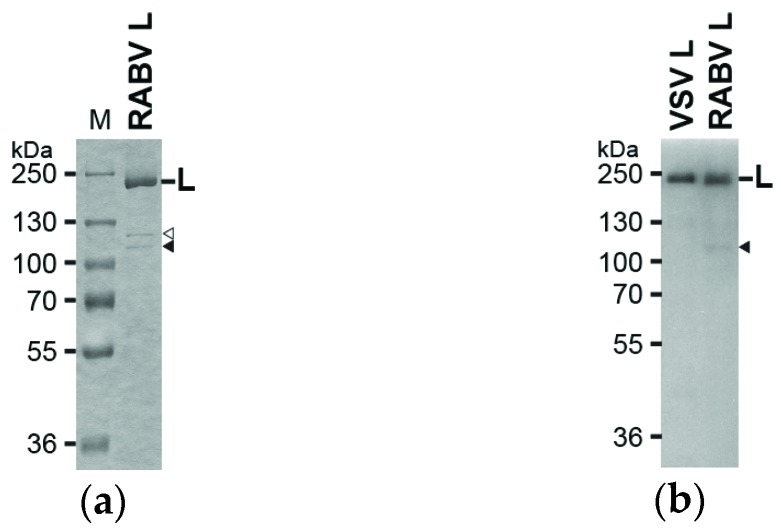
Sodium dodecyl sulfate polyacrylamide gel electrophoresis (SDS-PAGE) and Western blot analyses of the recombinant rabies virus (RABV) L protein: (**a**) The purified recombinant C-terminal His-tagged RABV L protein (0.7 μg) was analyzed by 7.5% SDS-PAGE followed by Coomassie Brilliant Blue staining. Lane M shows marker proteins with the indicated molecular masses. Open and closed arrowheads indicate minor proteins co-purified with the RABV L protein; (**b**) The recombinant vesicular stomatitis virus (VSV) and RABV L proteins (10 ng) were analyzed by Western blotting with anti-His antibody.

To investigate the putative cap-forming activities of the RABV L protein, we expressed and purified its C-terminal His-tagged form. The purified RABV L protein was analyzed by 7.5% SDS-PAGE followed by staining with Coomassie Brilliant Blue (Figure 1a). The full-length RABV L protein was co-purified with small amounts of proteins with molecular masses of 110 and 120 kDa, and, therefore, its purity was estimated to be ~70%. Western blotting with anti-His antibody suggested that the 110 kDa protein is a C-terminal fragment of the C-terminal His-tagged RABV L protein (Figure 1b, marked by a closed arrowhead).

**Figure 2:**
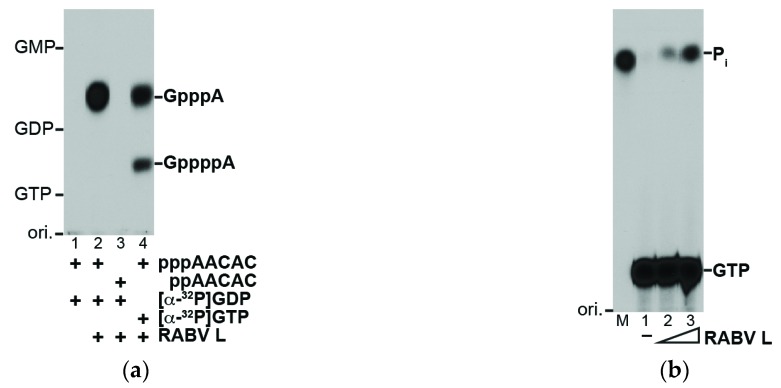
The RABV L protein generates the mRNA cap structure by an unconventional mechanism: (**a**) The recombinant RABV L protein (0.1 μg) was incubated with the indicated substrates under the standard conditions for the VSV L protein. Nuclease P_1_-digests of RNA products were analyzed by thin layer chromatography on a polyethyleneimine-cellulose plate (PEI-cellulose TLC), followed by autoradiography. The positions of the origin (ori.), guanosine nucleotides (GMP, GDP, GTP), and cap structures (GpppA, GppppA) are shown; (**b**) The recombinant RABV L protein (lane 2, 0.1 μg; lane 3, 0.2 μg) was incubated with [γ-^32^P]GTP. The reaction mixtures were analyzed by PEI-cellulose TLC followed by autoradiography. Lane 1 indicates no enzyme. Lane M shows the position of ^32^P-labeled inorganic phosphate (P_i_), which was generated by digestion of [γ-^32^P]GTP with alkaline phosphatase.

The purified recombinant RABV L protein was subjected to the VSV *in vitro* capping assay with a 5′-triphosphorylated oligo-RNA with a RABV mRNA-start sequence (5′-AACAC), instead of the VSV mRNA-start sequence (5′-AACAG) (Figure 2a). The RABV L protein capped 5′-triphosphorylated (lane 2) but not diphosphorylated (lane 3) AACAC with [α-^32^P]GDP to generate the G(5′)ppp(5′)A cap structure. The RABV L protein (0.1 μg) produced 0.91 ± 0.15 fmol (mean ± standard deviation of three independent determinations) of GpppA with GDP for 2 h. This result indicates that the specific activity (9 fmol GpppA/μg protein/2 h) of the RABV L protein is significantly lower than the reported specific activity of the VSV L protein (5 pmol GpppA/μg protein/2 h [27]), although these activities cannot be directly compared because of the results from independent experiments with the different RNA substrates for the respective L proteins. When using [α-^32^P]GTP as a substrate (lane 4), G(5′)ppp(5′)A (0.40 ± 0.04 fmol) and the tetraphosphate-containing cap structure G(5′)pppp(5′)A (0.19 ± 0.00 fmol) were produced with the RABV L protein as reported for the VSV L protein [17]. G(5′)pppp(5′)A appears to be formed as a by-product by the pRNA transfer to GTP rather than to GDP [17]. As expected, the RABV L protein hydrolyzed the γ-phosphate of GTP into P_i_ (specific activity, 0.6 pmol P_i_/μg protein/2 h) in a dose-dependent manner (Figure 2b, lanes 2 and 3). These results suggest that the RABV L protein catalyzes the unconventional capping reaction with the GTPase and PRNTase activities.

### 3.2. The RABV L Protein Specifically Caps the RABV mRNA-Start Sequence

**Figure 3:**
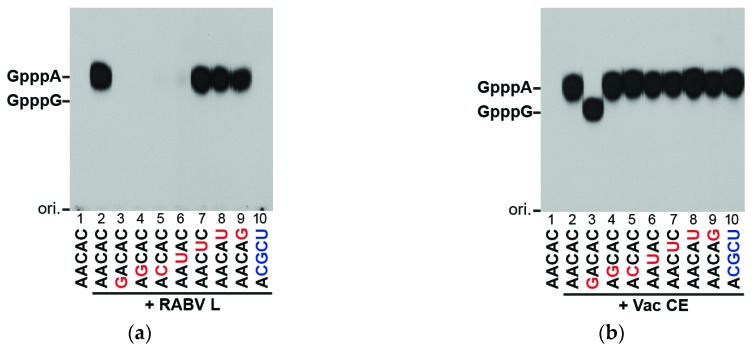
The RABV L protein caps virus-specific mRNAs: (**a**) The recombinant RABV L protein (0.1 μg) was incubated with [α-^32^P]GDP and 5′-triphosphorylated oligo-RNAs with the indicated sequences; (**b**) Vaccinia virus capping enzyme (Vac CE) was incubated with [α-^32^P]GTP and the RNAs. Nuclease P_1_-digests of RNA products were analyzed as in Figure 2. Lane 1 indicates no enzyme. The positions of the origin (ori.) and cap structures (GpppA, GpppG) are shown.

mRNAs of RABV and RABV-related lyssaviruses are known to start with the 5′-AACAY (Y, C or U) sequence which is similar to the mRNA-start sequence AACAG of vesiculoviruses [28]. To identify key nucleotide residues in the RABV mRNA-start sequence required for capping with [α-^32^P]GDP by the RABV L protein, we measured substrate activities of oligo-RNAs with various sequences (Figure 3a). Replacement of the first three nucleotides (A_1_, A_2_, and C_3_) in AACAC (lane 2) with different nucleotides abolished (lanes 3–5) or significantly diminished (lane 6) their substrate activities, while replacement of A_4_ and C_5_ showed no or modest effects on their activities (lanes 7–9). As expected, the RABV leader RNA-start sequence, ACGCU, did not serve as a substrate for the RABV capping enzyme (lane 10). In contrast, the conventional mRNA capping enzyme of vaccinia virus efficiently capped all these RNA substrate with [α-^32^P]GTP (Figure 3b, lanes 2–10). These results indicate that the RABV L protein strictly recognizes the first three nucleotides of the RABV mRNAs to form the cap structure.

### 3.3. Conserved Amino Acid Residues in PRNTase Motifs A–E of the RABV L Protein are Required for mRNA Cap Formation

**Figure 4:**
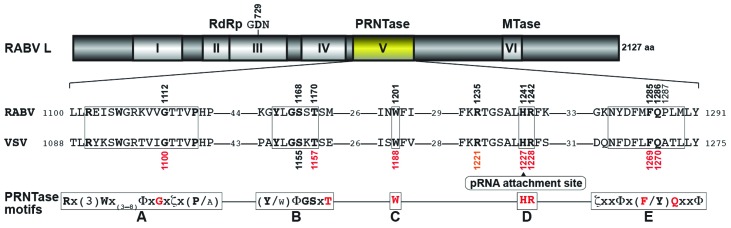
The polyribonucleotidyltransferase (PRNTase) motifs in the RABV L protein: A schematic structure of the RABV L protein is shown with six conserved amino acid sequence blocks. RdRp and MTase indicate RNA-dependent RNA polymerase and methyltransferase, respectively. Local amino acid sequences of the putative PRNTase domain (block v) of the RABV L protein were aligned with those of the VSV L protein. The numbers above and below the sequences indicate the positions of amino acid residues in the L proteins of RABV and VSV, respectively. H1227 in motif D of the VSV L protein has been identified as the covalent pRNA attachment site [19]. Consensus amino acid sequences of the PRNTase motifs A–E [22] are shown on the bottom (x, Φ, and ζ indicate any, hydrophobic, and hydrophilic amino acids, respectively).

Using the recombinant VSV L protein, we have previously identified key amino acid residues in PRNTase motifs A–E that are required for RNA capping in the step of the L-pRNA intermediate formation [19,22]. All the motifs are strictly conserved in the L proteins of RABV (Figure 4) and other lyssaviruses [22].

**Figure 5:**
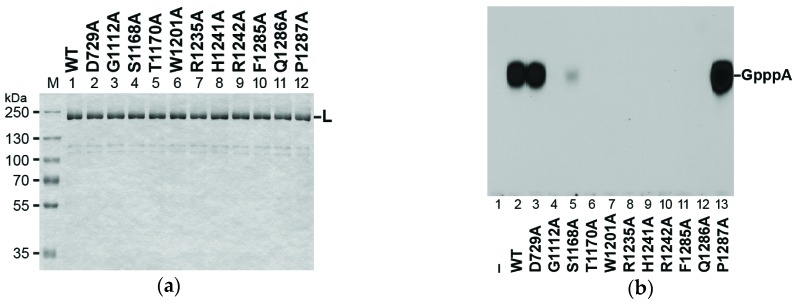
The PRNTase motifs are essential for the RNA capping activity of the RABV L protein: (**a**) The wild-type (WT) and mutant RABV L proteins (0.7 μg) were analyzed by SDS-PAGE as described in Figure 1a; (**b**) The WT and mutant RABV L proteins (0.1 μg) were subjected to *in vitro* capping reactions with [α-^32^P]GDP and pppAACAC. Lane 1 indicates no enzyme.

In order to investigate whether these motifs participate in RNA capping with the RABV L protein, we generated L proteins with alanine mutations in selected residues. Similar to the WT L protein (Figure 1a; Figure 5a, lane 1), all these mutant L proteins were co-purified with small amounts of the 110 kDa and 120 kDa proteins (Figure 5a, lanes 2–12). As shown in Figure 5b, while the D729A mutation in the RdRp active site [29] did not affect the capping activity of the RABV L protein (lane 3), mutations in the PRNTase motifs, G1112A (lane 4) in motif A, T1170A (lane 6) in motif B, W1201 (lane 7) in motif C, H1241 (lane 9) and R1242 (lane 10) in motif D, and F1285 (lane 11) and Q1286 (lane 12) in motif E, abolished the capping activity. Similar to the S1155A mutation in motif B of the VSV L protein [22], S1168A (lane 5) significantly reduced the capping activity of the RABV L protein to 9% of the WT activity.

Using the VSV and CHPV L proteins, we have previously identified a rhabdovirus-specific arginine residue (R1221 for VSV; R1211 for CHPV) in the vicinity of motif D as essential for the PRNTase activity [18,19]. Consistent with these findings, the RABV L protein with an alanine substitution (R1235A) for the counterpart of R1221 of the VSV L protein was found to be inert in RNA capping (lane 8). Interestingly, the alanine mutation in the non-conserved P1287 residue in motif E increased the capping activity 3.6-fold (lane 13). It should be noted that the VSV L protein has an alanine residue at this position in motif E (see Figure 4). Taken together, these results demonstrated that the PRNTase motifs in the RABV L protein are essential for the capping activity.

## 4. Discussion

Earlier studies reported that RNA synthesis activities of RABV ribonucleoproteins and detergent-disrupted virions are significantly lower than those of VSV [5,6]. Therefore, it has remained challenging to further characterize RABV enzymes involved in mRNA biogenesis using *in vitro* transcription systems. Here, we demonstrated that the purified recombinant RABV protein catalyzes the unconventional RNA capping reaction with the PRNTase activity, although to a much lesser degree than the VSV L protein. This is the first example to directly show the enzymatic activities of the RABV L protein *in vitro*, thus opening up the opportunity to investigate the functions of this poorly characterized protein.

**Figure 6:**
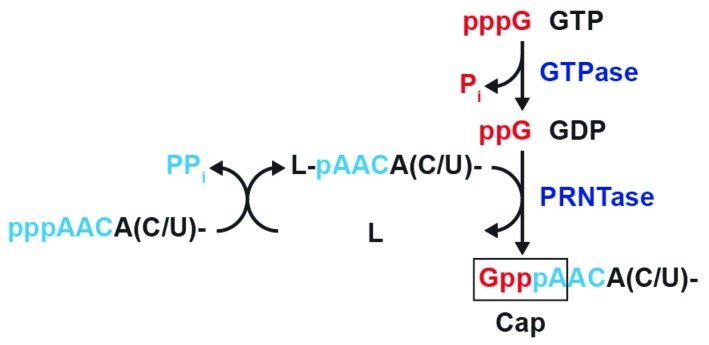
A proposed pathway of RABV mRNA capping: The RABV L protein–associated guanosine 5′-triphosphatase (GTPase) activity hydrolyzes GTP into GDP. The PRNTase domain in the L protein transfers pRNA from pppRNA to GDP via a putative covalent L-pRNA intermediate to produce capped RNA.

The recombinant RABV L protein specifically capped pppRNA, but not ppRNA, with GDP, generated from GTP with GTPase (Figure 2), as reported for the VSV L protein [16]. In contrast, mRNA guanylyltransferases (GTase) of eukaryotes, nucleocytoplasmic large DNA viruses (e.g., vaccinia virus), and double-strand RNA viruses are known to use GTP, but not GDP, and ppRNA, generated from pppRNA with RNA 5′-triphosphatase, as substrates to generate the G(5′)ppp(5′)N cap structure [30,31,32]. Thus, as in the case of the VSV L protein [16,19], the RABV L protein was strongly suggested to transfer pRNA from pppRNA to GDP via a covalent L-pRNA intermediate with the PRNTase activity (Figure 6). However, so far, our attempts to demonstrate the formation of the putative intermediate with the RABV L protein have failed because of the low specific activity of the PRNTase domain.

Similar to the recombinant VSV L protein as well as the native VSV L-P RdRp complex [16,17], the GTPase activity was co-purified with the recombinant RABV L protein (Figure 2b). This activity is required to hydrolytically convert GTP into GDP, which acts as the pRNA acceptor for the following PRNTase reaction (Figure 6). Although some mutations (e.g., Y1152A in motif B, W1188F in motif C) in the PRNTase domain of the VSV L protein were found to abolish and diminish the PRNTase and GTPase activities, respectively [22], it still remains unclear whether the L protein itself catalyzes GTP hydrolysis. Further studies are needed to identify the putative GTPase domain/active site in the rhabdoviral L proteins or possibly cellular GTPase associated with these L proteins.

As reported for the recombinant VSV L protein [17], the recombinant RABV L protein produced the G(5′)pppp(5′)A cap structure on RNA, probably as a by-product when using GTP as a substrate (Figure 2a). G(5′)pppp(5′)A seems to be generated by the transfer of pRNA from pppRNA to GTP prior to hydrolysis of GTP into GDP under the optimal conditions for the PRNTase activity, but not for the GTPase activity. Although G(5′)pppp(5′)A is known to be formed on a small part of mRNAs synthesized by native VSV ribonucleoproteins *in vitro* [17], its biological significance, if any, is not known.

We have previously reported that the VSV L protein efficiently caps pppRNAs with the VSV mRNA-start sequence (5′-AACAG). In this sequence, the A residue at position 1 (A_1_) and the pyrimidine residue at position 3 (Y_3_, C > U) are critical for the substrate activity with VSV PRNTase, but the A residue at position 2 (A_2_) can be functionally replaced with G or partially with C [16,17]. Since the mRNA-start sequences of RABV and RABV-related lyssaviruses (5′-AACAY) are very similar to that of VSV [28], lyssaviral L proteins were suggested to recognize their mRNA-start sequences with VSV-like RNA-binding sites in their PRNTase domains [16]. In fact, similar to VSV, the A_1_ and C_2_ residues in pppAACAC were found to be essential for RNA capping with the RABV L protein (Figure 3). To our surprise, however, we found that the A_2_ residue in pppAACAC cannot be substituted with G or C for RNA capping with the RABV L protein. These results suggest that the RABV PRNTase domain recognizes the 5′-AAC sequence more strictly than VSV.

We verified that conserved amino acid residues (G1112, T1170, W1201, H1241, R1242, F1285, and Q1286) in the PRNTase motifs A to E of the RABV L protein are critical for RNA capping (Figure 5). Significant homology of the putative PRNTase domain in the RABV L protein to that in the VSV L protein (Figure 4) suggests that these residues constitute the active site of the RABV PRNTase domain and are required for the L-pRNA intermediate formation in the pRNA transfer reaction.

H1241 in motif D (HR motif), the counterpart of H1227 of the VSV L protein, most probably serves as a nucleophile to form the putative L-pRNA intermediate. For the L-pRNA intermediate formation, the basic amino acid residue R1242 in motif D may interact with the 5′-triphosphate group of pppRNA and facilitate the release of PP_i_, possibly by acting as a proton donor. Other residues in motifs A, B, C, and E appear to be involved in pppRNA binding and/or structural maintenance of the PRNTase domain. Although Li *et al.* [23] reported that G1100 in motif A of the VSV L protein is not required for mRNA capping, our recent [22] and current (Figure 5) studies showed that G1100 of the VSV L protein and its RABV counterpart, G1112, play an important role in mRNA capping.

The arginine residue (R1235 for RABV; R1221 for VSV; R1211 for CHPV), six amino acids upstream of the catalytic histidine residue (H1241 for RABV; H1227 for VSV; H1217 for CHPV), is conserved only in the L proteins of some rhabdoviruses (e.g., lyssaviruses, vesiculoviruses, ephemeroviruses, spriviviruses, perhabdoviruses) that possess the 5′-AAC mRNA-start sequence [9,18,19,24]. In contrast, L proteins of other nonsegmented negative-strand RNA viruses having different sets of mRNA-start sequences (e.g., 5′-AGG, 5′-AGA, 5′-GGG, 5′-GAA, 5′-GAU) do not possess an arginine residue at this position [9,19,24]. One possibility is that this arginine residue is involved in the recognition of the C_3_ residue in pppAAC-RNA.

In this study, using our *in vitro* RNA capping assay with the recombinant RABV L protein, we demonstrated that the RABV L protein catalyzes the unique RNA capping reaction with the putative PRNTase domain in an mRNA-start sequence–dependent manner. Studies are under way to further characterize the mRNA capping activity and other enzymatic activities, such as RNA synthesis and cap methylation activities, of the RABV L protein. *In vitro* RNA synthesis and processing systems with the recombinant RABV L protein will be useful for elucidating the molecular mechanisms of lyssaviral mRNA biogenesis and developing antiviral agents against lyssaviral L proteins.

## Abbreviations

The following abbreviations are used in this manuscript:

RABV	rabies virus
VSV	vesicular stomatitis virus
CHPV	Chandipura virus
PRNTase	polyribonucleotidyltransferase
GTPase	guanosine 5′-triphosphatase
RdRp	RNA-dependent RNA polymerase
SDS-PAGE	sodium dodecyl sulfate polyacrylamide gel electrophoresis
PEI-cellulose TLC	thin layer chromatography on a polyethyleneimine-cellulose plate
WT	wild-type
